# Sexual Behaviour and Attitudes towards Safe Sex of Youth Receiving Antiretroviral Care at Public Health Facilities in Palapye District, Botswana

**DOI:** 10.3390/ijerph20053790

**Published:** 2023-02-21

**Authors:** Onai Diura-Vere, Mathildah M. Mokgatle, Oluwafemi O. Oguntibeju

**Affiliations:** 1 Department of Public Health, School of Health Care Sciences, Sefako Makgatho Health Sciences University, Ga-Rankuwa 0208, South Africa; 2 Department of Biomedical Sciences, Faculty of Health and Wellness Sciences, Cape Peninsula University of Technology, Bellville 7535, South Africa

**Keywords:** sexual behaviour, HIV-infected youths, attitudes towards safe sex, risky sexual behaviours, Botswana

## Abstract

Background: Sexual behaviour of HIV-infected youths is very important in determining the direction of the HIV epidemic, as these youths are reservoirs of HIV and can propagate its transmission if they engage in risky sexual behaviours. However, support structures for secondary prevention are weak even in healthcare settings. There is a need to understand the sexual behaviour of these youths and, in turn, tailor appropriate secondary prevention strategies, hence the current study was designed to assess sexual behaviour and attitudes towards safe sex of youth receiving antiretroviral care at public health facilities in Palapye district, Botswana. Method: This quantitative, descriptive cross-sectional survey was used to describe the sexual behaviour and attitudes towards safe sex and identify factors associated with risky sexual behaviours among HIV-infected youths aged between 15 and 19 years receiving antiretroviral therapy (ART) care from public healthcare facilities in Palapye District, Botswana. Results: A total of 188 youths participated in this study, 56% being females while 44% were males. We reported that 15.4% had ever had sex. At their last sexual encounter, more than half of the youths (51.7%) had not used condoms. More than a third of the participants were under the influence of alcohol during their last sexual experience. Generally, the youths had good attitudes towards safe sex, as most youths said they would prioritise protecting their sexual partners and themselves from HIV and STIs. Alcohol use, substance use and not considering religion as important were strongly associated with having ever had sex. Conclusions: A significant proportion of HIV-infected youths are sexually active, whereas their preventive practices such as condom use are poor despite good attitudes towards safe sex. Alcohol use, substance use and not perceiving religion as important were associated with risky sexual behaviours.

## 1. Introduction

The number of children with perinatal human immunodeficiency virus (HIV) infection surviving into adolescence and young adulthood has been increasing [1,2,3]. This has been linked to the successful roll-out of antiretroviral therapy (ART) in most parts of the world including Sub-Saharan Africa. Globally, there are 1.6 billion people aged 12–24 years, the largest generation of adolescents and young people ever [4,5]. Eighty-two percent of the estimated 2.1 million adolescents aged 10–19 years living with HIV are believed to be resident in Sub-Saharan Africa [5].

In the transition from childhood to adulthood, adolescents present with multiple challenges and experimental behaviours [1,2,3,4,5]. HIV infection further adds complexity to this stage of life which often includes initiation of sexual activity [6,7]. The common characteristics observed during this stage include exposure to health risks such as alcohol use and risky sexual behaviour [4,5,6]. By the time the youths start to develop intimate relationships, they are already HIV-positive and are usually aware of their status. HIV-infected youths, therefore, face early and foreseen challenges with HIV status disclosure, negotiating safe sex and adherence to ART [7]. Poor sexual behaviour poses a health risk to these youths as they risk super-infection, infection with other sexually transmitted infections (STIs) and propagation of HIV transmission to their uninfected sexual partners. These risks are further escalated if these youths fail to disclose their status and adopt safer sexual behaviours such as consistent condom use [6,7]. Despite all these challenges, the sexual and reproductive health needs of this unique and growing population are largely unmet [8,9,10] and research on this new cohort is also limited.

Sexual behavior has implications for individual wellbeing, public health, fertility and other important phenomena [11]. There are several determinants of sexual behavior and initiation of sexual activity before marriage. The most important ones based on literature are individual characteristics such as onset of puberty, age, race, socio-economic status, religion, intelligence, and academic achievement, dating behavior, family characteristics such as family background and parental support and control and influence of peer groups [12,13]. Social networks also played a major role in shaping numerous specific sexual behaviors, notably sexual intention, sexual initiation, sexual orientation and identity, sexual frequency, sex partner selection and sex markets and risky sexual behaviors [8,9,10,13]. Several cross-sectional and longitudinal studies across Africa have attempted to describe and explore sexual behavior of adolescents and young adults in different settings. The settings range from communities where out of school youths were targeted, schools, colleges and universities and healthcare settings [11]. The findings have seen a consistently high prevalence of sexual activity increasing with age [11,12,13]. Because sexual behavior is culture- and environment-specific, a systematic review conducted in 24 Sub–Saharan African countries provides standard baseline data on youth sexual behavior in Sub-Saharan Africa (SSA). It involves adolescents who were particularly aged between 15 and 19 years old and sought to describe the sexual and reproductive behavior of these youths. Important findings for Southern Africa were that a higher proportion of males were sexually active before the age of 15 years compared with girls. Condom use at last sex within non-marital relationships remained low with figures below 50% in most countries. Males were, however, more likely to use condoms compared with females. A significant proportion of males reported multiple sexual partners compared with females [14,15,16].

Botswana, like other countries, has come up with a life skills school HIV curriculum to empower youths with HIV-related knowledge. However, most of the messages are aimed at developing skills for primary HIV prevention whereas HIV-positive youths would benefit more from secondary prevention messages which are visibly weak in the school curriculums [15]. In public healthcare facilities where such programmes can be rolled out, there is no designated organised and coordinated public sector adolescent care in place [15]. Most specialised care is targeted at paediatric care and adult care. Teen clubs for HIV-infected adolescents who receive care in some public facilities have been established in some few districts but are not fully functional due to inadequate support and funding. Botswana has moved towards the ‘Universal Treat All’ strategy, which was launched in June 2016 as a way of helping to achieve epidemiological control of HIV [15]. This relies on the assumption that once all willing HIV-infected individuals are initiated on ART as soon as possible after diagnosis regardless of their CD4 count, and are virally suppressed, the chance of transmitting HIV greatly diminishes resulting in reduction in HIV transmission rates both horizontally and vertically. However, this may not apply to HIV-infected youths. Based on a systematic review by Kim et al., and a nationally representative survey by Zhou et al. [17], almost 40% of adolescents who are eligible for or have started ART are non-adherent to treatment. Non-adherence to ART results in detectable viral loads which can be picked during routine laboratory monitoring. Reports from several Sub-Saharan countries also suggested that children and adolescents on ART are almost twice as likely to have virologic failure compared with adults [17,18,19]. This has implications for HIV transmission including the possibility for sexual transmission of ART drug resistant strains of HIV [6,7,17,20]. It is the belief of the authors that safer sexual behaviours remain a very important HIV prevention strategy in this unique population group. The concerns around the high incidence of HIV, risk for further HIV transmission and possibility of transmission of resistant strains of HIV and vulnerability to HIV re-infection among HIV-positive youths against weak support facilities prompted this study. The study seeks to understand the sexual behaviours, attitudes towards safe sex and factors that are associated with risky sexual behaviours among youths on ART in Palapye District as a way of identifying key issues and providing evidence for secondary prevention programmes.

Findings from this current study would help modify the current organisation and package of adolescent HIV care being offered at public healthcare facilities in Palapye and Botswana with more emphasis on empowering these youths towards achieving sexual health. Understanding sexual behaviour and risky sexual behaviours of youths living with HIV/AIDS is critical to secondary prevention HIV. Studying the sexual behaviour, attitudes towards safe sex and factors associated with risky sexual behaviours among HIV-infected adolescents in Palapye District would help inform and tailor programmes for prevention of secondary HIV transmission most suited for Palapye and probably Botswana youths within the context of their culture and environment. This is because sexual behaviour is culture and environment specific and interventions should be developed within a culturally sensitive framework to optimise care [18,21]. Empowering HIV-positive adolescents with skills to achieve sexual health would not only result in their improved wellbeing but would help prevent further HIV transmission, which is a current major public health concern.

## 2. Materials and Methods

### 2.1. Study Design

A non-experimental (observational), quantitative, descriptive cross-sectional survey approach, was used in this study targeting HIV-infected youths aged between 15- and 19-years receiving ART care from public healthcare facilities within Palapye DHMT, Botswana. The survey data was obtained using a structured interviewer administered questionnaire. Pertinent independent variables included socio-demographic factors such as age, gender, religious affiliation, level of education, caregiver status, communication with important others about sexual health, alcohol and drug use. Important dependent/outcome variables were ever having had a boyfriend or girlfriend, being in a current relationship, ever having had sex, having been pregnant or having had impregnated a girl, risky sexual behaviours such as having multiple sexual partners and poor condom use patterns and non-disclosure. Due to response rate issues, a census of all 338 HIV-infected adolescents receiving ART was chosen as the sampling technique. They were recruited in the healthcare facilities as they came for their routine care appointments. Data was captured using Excel and analysed using STATA version 13.

### 2.2. Study Population

The study population was all HIV-infected youths aged between 15 and 19 years currently registered and receiving ART care in public health care facilities within Palapye DHMT of Botswana.

### 2.3. Sampling and Recruitment

From a target population of N = 338 youths who were enrolled in ART care in the public healthcare facilities, convenient sampling technique was employed and the sample size was a census of adolescents who consented and/or assented to participate in the study. The rationale for the convenient sampling technique and the census sample size was to accommodate refusal and non-response from a small population size. Refusal of consent from some parents of the youths who were under the age of 18 years was also considered. Some youths under the age of 18 years who reported for care unaccompanied by parents or caregivers were not recruited for ethical reasons.

Participants were recruited from all health care facilities offering ART prescription services which are Palapye Primary Hospital and nine clinics. Youths aged between 15 and 19 years old who were visiting these facilities for their routine reviews, medication refill appointments and those coming for laboratory services during the period of data collection were recruited. Recruitment took place after the nurse had taken vital signs upon arrival at the ART service points before or after they had received their routine care.

### 2.4. Inclusion and Exclusion Criteria

Youths aged between 15 and 19 years who receive ART from the selected facilities who volunteered to participate in the study and gave consent or assent were included in the study. Those who did not give consent and those who had not been disclosed to or were not aware of their HIV-positive status were excluded from the study.

### 2.5. Data Collection Tool

An interviewer administered structured questionnaire in English and a translated local Setswana language was used. It was structured to limit the responses to only those that were useful for the study and make the data easy to analyse. The questionnaire was developed to enable the collection of information that would ensure that the study questions are eventually answered and that the study objectives are met. The questionnaire was designed guided by literature and partly adapted from a standardized questionnaire on “Asking young people about sexual and reproductive behaviour” by [22]. Using a standardised questionnaire helped ensure validity.

The questionnaire had four sections. The first section (section A) captured the socio-demographic characteristics of the participants including their sex, age educational background, employment status (where applicable), religion and its importance to the participant’s life, caregiver status and social behaviours such as smoking, alcohol intake and drug use.

Section B captured the general reproductive health context of the youths. This included whether they talked about sex, had reproductive health knowledge and their preferred source of such information and these questions were also guided by literature.

Section C comprised 20 questions relating to sexual behaviour. Such questions included sexual partner preference, ever had, and having a current boyfriend or girlfriend, HIV status disclosure, having ever had sexual intercourse, number of sexual partners and age at first sex. The next questions sought to explore preventive practices such as condom use, alternative contraception use at first and last sex and consequences of unprotected sexual intercourse resulting in pregnancy. Engagement in same sex sexual intercourse, anal sex, group sex and sex under the influence of alcohol were also asked, as being some of the risky sexual behaviours according to literature.

Section D consisted of 11 questions pertaining to attitudes towards safe sex. A 3 point-Likert scale [22] with agree, neither agree nor disagree and disagree was found to be more reliable and could easily translated to Setswana without losing meaning and was also found to be easier for the target respondents to understand. This was also confirmed during the pilot. Respondents were asked to indicate their extent of agreement or disagreement on a 3-point scale to each of the 11 statements concerning safe sex practices.

### 2.6. Data Collection

The questionnaire was administered by the study team in Setswana and English. The questionnaire was interviewer administered to minimize missing data by making sure that all questions had been responded to. The interviewer would also clarify participants’ questions, resulting in more accurate responses. Asking questions about sexual behaviour is particularly sensitive, one-on-one interviews offer the greatest confidentiality and may provide the most candid answers [23]. The counselling room for each respective facility was used for data collection to provide privacy which the study required. A pilot study with 10 participants was conducted to test the different aspects of the study methodology before the actual data collection. Data from the pilot was subsequently included in the main study due to the low response rate. The participants of the pilot study were also part of the main study. Permission to conduct the study was granted by the Bostwana Ministry of Health, Health Research and Development Division, on 20 September 2016. Data was collected from October 2016 to February 2017.

### 2.7. Data Analysis

Data was captured in a Microsoft Excel spreadsheet, validated, and coded. The data was imported into STATA version 13 for analysis. Independent variables in this study are the socio-demographic factors such as age, gender, religious affiliation, level of education, caregiver status, communication with important others about sexual health, alcohol, and drug use. Dependent/outcome variables are having had sex, having been pregnant or having had impregnated a girl, risky sexual behaviours such as having multiple sexual partners and poor condom use patterns. Descriptive statistics were used to summarise demographic variables such as age, gender, and level of education. Means and standard deviations were calculated for continuous variables such as age of the participant, age at first sex and number of sexual partners. Frequencies and cross tabulations were calculated for categorical variables. Associations were computed using bivariate analyses based on chi square and *p* value. Logistic regression was used to determine the factors associated with risky sexual behaviours: the odds ratio, *p* values and confidence intervals.

### 2.8. Reliability and Validity

To improve validity, a standardized questionnaire on “Asking young people about sexual and reproductive behaviour” by [19,24] was adopted. The standardised tool also improved reliability. Field workers were trained on administering the questionnaire to ensure that all questions were asked in a similar and standardized manner. During the administration of the questionnaire, participants were encouraged to respond honestly and due to the sensitive nature of an HIV-positive status and sexual behaviour, maximum privacy possible was offered to encourage honest responses.

### 2.9. Ethical Considerations

Ethical clearance was obtained from the School of Health Care Sciences Ethics Committee and the Sefako Makgatho Health Sciences University Research Ethics Committee (SMUREC). Permission to conduct the study was also obtained from Ministry of Health Botswana Research Board and Palapye District Health Management Team. Permission to collect data was verbally sought from Chief Medical Officer Palapye Primary Hospital and Nurses in charge of local clinics. Participation in the study was voluntary. Written informed consent was obtained from participants 18 years and older. Assent and parental/guardian consent was obtained from adolescents below 18 years prior to their participation in the study. This involved giving information about the nature of the research project, potential risks and how their participation would contribute to the study. Participants were also informed that their decision not to participate or even to withdraw would not affect further care. They were assured of confidentiality and anonymity which meant that the information they gave would not be identified as theirs. Before participation in the study, the nurse informed the youths and their caregivers about the study and once they showed interest to participate, they were referred to a member of the study team who was typically in a separate private room, usually the facility’s counselling room. The purpose of the study and other study modalities were explained to the participants and the willing participants and caregivers gave consent and or assent prior to questionnaire administration. Field work was performed in a manner that minimized disruptions in service provision.

### 2.10. Study Setting

Palapye is one of the districts in the greater central district of Botswana. It is classified as a village but has been growing rapidly into an urban centre, with ‘suburbs’ rapidly being established. Palapye District has an estimated population of 90,000, based on the 2011 census and population projections. The region has grown chiefly due to its central location, the Morupule Coal Mine and the power station which has been recently upgraded. Health care services in Botswana, Palapye included are mainly provided by the Government through the Ministry of Health and Wellness under Palapye District Health Management Team (DHMT). Palapye District has one primary hospital, nine clinics and twenty-one health posts. The hospital acts as the referral centre for all the clinics and health posts within the district. However, only the hospital and clinics currently offer ART prescription services.

## 3. Results

### 3.1. Participants Characteristics

A total of 188 (n = 188) HIV-infected youths aged between 15 and 19 years participated in this study. Fifty-six percent (n = 106) were females whilst 44% (n = 82) were males. The mean age was 17 years old, (SD: 1.43). All participants except for one had attended school. Most of the youths had reached secondary school (82%, n = 155), 14% were at primary school, 5% had completed vocational training and 1% certified training. Sixty eight percent (n = 126) were in school whilst 32% (n = 62) were out of school. Of the out of school youths, only 22.5 % were employed (Table 1).

The majority (85.1%, n = 160) reported that they were Christians and most youths also reported that religion was important to them. Approximately a third of participants were staying with the mother (38.3%, n = 72), 12% stayed with both parents whilst 10% stayed with the father. A small percentage of these youths were staying alone (2.7%, n = 5) whilst the remaining 40% stayed with a grandparent, sibling or any other caregiver. The caregiver status was further categorised into two distinct categories, those that stayed with one or both biological parent and those that stayed with other caregivers. Only 2 % of the youths had used habit-forming drugs and these were only males. Approximately 4 % smoked cigarettes, with more males reporting smoking compared with females and 14% drank alcohol (Table 1).

### 3.2. Basic Reproductive and Social Context of the Participants

Table 2 presents the basic reproductive health and social context of the participants. Some of the questions posed to the participants were questions relating to who they talk to about important and private things, if they ever talk about sex and how easy is it to talk about sex. Data shows that about a third of the youths spoke to their parents, especially mothers, about important things. The remainder spoke either to grandparents, other relatives, siblings, friends, and teachers. About 12.2 % (n = 23) of the youths (participants) kept things to themselves and these were mainly males (Table 2).

Approximately two thirds had discussed sex-related issues with some of the people referred to above. A third had found it easy to discuss, another third said it was average whilst the remaining third found it difficult. Almost 90% of the youths reported that they had some knowledge pertaining to reproductive health although the kind and extent of the knowledge was not assessed. This reproductive health knowledge had been acquired mainly from their school teachers (n = 61; 32.4%). The second most important source were healthcare workers (13.8%), followed by media (12.2%). Parents, other relatives, books, the Internet, church, siblings and friends were also cited as sources (Table 2). The majority of the youths (n = 64; 34%), however, preferred to receive reproductive health information from healthcare workers. Some also preferred to receive this information from the media (n = 33; 17.6%), their mothers (n = 21; 11.2%), teachers (n = 24; 12.7%), and at church (n = 12; 6.4%).

Although most of these youths (44.4%), said they preferred a partner who is also HIV-positive, 17% said they preferred an HIV negative partner. Thirty-nine percent did not mind their partner’s HIV status and would have a partner with any HIV status.

### 3.3. Sexual Behaviour: Relationships and Disclosure

Forty-five (45; 24%) reported having ever had a boyfriend or a girlfriend, with n = 33 (17.6%) having a current boyfriend or girlfriend. HIV status disclosure was assessed for all respondents who had reported that they had ever had boyfriends or girlfriends. Of those youths that had had boyfriends or girlfriends, only 36% (n = 16) had disclosed their HIV-positive status with a higher proportion of females disclosing (42%) compared with males (26%).

### 3.4. Sexual Experiences

It was reported that 15.4%, (n = 29) had sexual intercourse, which was defined as vaginal, oral or anal penetration. Seven girls had been pregnant, and two boys had impregnated girls, bringing the number of youths who had had pregnant-related outcomes to nine (31%) of the ever had sex youths and 5% of the study sample. More than half of the youths, n = 16 (55%) had experienced sexual intercourse within the three months prior to the study. Furthermore, more than a third n = 12 (40%), were under the influence of alcohol during their last sexual experience. There was no record of same sex-sex and group sex. Only one male participant reported having had unprotected anal sex.

### 3.5. Multiple Concurrent Partnerships

Among those who had sexual partners, the number of partners ranged from one to four. Most youths n = 15 (54%) reported only one sexual partner, whilst n = 8 (29%) had two partners. Four respondents (14%) had no current sexual partners and only 1 youth (4%) had 4 sexual partners, which was the highest number of sexual partners reported for this study population.

### 3.6. Age at First Sex

Age at first sex ranged from 11 to 19 years and the mean age at first sex was 16.3 years, 95% CI 15.7–16.9. The difference between the mean age at sexual debut between males and females was not significant (*p* = 0.324) based on an unpaired t-test.

### 3.7. Unprotected Sexual Intercourse/Condom Use Practice

Almost three quarters of the first sexual encounters were unplanned and only 14% had used any method of contraception besides a condom. Still, during their first sexual encounters, less than half (45%) n = 13 youths had used condoms. At last sex, condom use remained below 50% (41%) n = 14, with more female study participants reporting condom use compared with the males (Table 3). In terms of the overall condom use practice, only about a quarter reported consistent condom use which was represented by the phrase “I always use condoms”. The majority, n = 21 (70%), however, reported inconsistent condom use which was represented by the phrase “I sometimes use condoms”. The difference in condom use practice between males and females in this study was, however, not significantly different (*p* = 0.555).

### 3.8. Attitudes towards Safe Sex

To test the attitudes of the participants towards safe sex, the respondents were given 11 statements partially adapted from the Illustrative Questionnaire for Interviews-Surveys with young people (Cleland, 2011). They reported their agreement or disagreement on these attitude indicators on a three-point scale ranging from agree, not sure and disagree.

Overall, the respondents expressed positive attitudes towards safe sex (Table 4). More than half of the youths n = 105 (56%), believed it was alright for youths of the opposite sex to hug, touch and kiss. Similarly, n = 99 (53%) believed there was nothing wrong with premarital sex among youths if they love each other. However, n = 78 (41%) would refuse to have sex with a partner who was not prepared to use a condom.

About three quarters of the respondents disagreed with one-night stand. Despite the finding that 55% reported that it was not embarrassing for them to buy or obtain condoms; a third (32%) felt it was embarrassing to buy or obtain condoms. Almost two thirds disagreed with the statement that when a girl suggests condom use it meant that she did not trust her partner. The majority, n = 136 (72%) believed that condoms could protect their sexual partner from HIV infection. Eighty-four and 91% of the youths would prioritise protecting their partner from being infected with HIV and prioritise themselves from super-infection and STIs, respectively.

### 3.9. Factors Associated with Risky Sexual Behaviour

A bivariate analysis of risky sexual behaviours against socio-demographic characteristics and reproductive health and social context was performed. The risky sexual behaviours that were found to have significant relationships with socio demographic characteristics and reproductive health and social context and were therefore selected include: having ever had sex, condom use practice and non-disclosure of an HIV-positive status (Table 5). Having ever had sex was put as dependent variables on logistic regression model and the results are summarised in Table 6. An attempt to put condom use practice as a dependent variable in a logistic regression model failed due to an insufficient sample size of sexually active youths, which was only 29.

### 3.10. Risky Sexual Behaviours: Ever Had Sex

The relationships between age of the respondent, whether the respondent was in school or out of school, importance attached to religion, alcohol use, smoking, substance use and self-reported reproductive health knowledge and having ever had sex were significant (*p* < 0.005) based on the bivariate analysis using the chi-squared test (Table 5), suggesting a relationship or association. Being older (age above 17 years) was associated with having ever had sex or initiation of sexual activity. The relationship between being in school or out of school and having ever had sex was also significant (*p* = 0.000). The relationship between the importance of religion and having ever had sex was also significant (*p* = 0.001). Alcohol use, smoking and substance use also had significant relationships with having ever had sex; *p* = 0.000, 0.000 and 0.001, respectively. Not having reproductive health knowledge was also associated with having ever had sex (*p* = 0.000).

However, when the above socio-demographic factors were put in a regression model with having ever had sex as the dependent variable, alcohol use was strongly associated with having ever had sex (*p* = 0.000; OR-12.5). Youths that reported alcohol use were 12.5 times more likely to have ever had sex compared to those that did not use alcohol. There was a strong association between having ever had sex and religion. Youths who felt religion was not important to them were almost 6 times more likely to have had ever had sex compared with those that felt religion was important (*p* = 0.005, OR-5.9). Substance use was also associated with having ever had sex (*p* = 0.390; OR-5.8). Youths who had used substance abuse were 5.8 times more likely to have had sex than youths who had not used substances.

### 3.11. Condom Use Practice

Condom use at first sex and condom use at last sex were excluded even though they also had significant associations with certain socio demographic and reproductive health and social context characteristics as they gave information almost like condom use practice. Condom use practice used to represent overall condom use by the respondents initially with three possible responses which were “I always use condoms”, “I sometimes use condoms” and “I never use condoms”. No respondent reported that they never use condoms, hence only the two earlier responses were considered inconsistent condom use, represented by “I sometimes use condoms” represented the risky sexual behaviour. The relationships between the importance attached to religion, alcohol use, having ever discussed sex-related issues and self- reported reproductive health and condom use practice were also significant (*p* < 0.005) based on the bivariate analysis using the chi-squared test, suggesting an association (Table 5). However, an attempt to put condom use practice as a dependent variable in a logistic regression model failed due to an insufficient sample size of sexually active youths which was only 29.

### 3.12. Non-Disclosure

Based on the bivariate analysis, the relationship between HIV status disclosure and having ever discussed sex-related matters (*p* = 0.033) and having sexual and reproductive health (*p* = 0.020) were significant. However, the factors were too few to be put in a logistic regression model.

## 4. Discussion

Sexual behaviour, especially among the youth, is important in determining the direction of the HIV pandemic due to its predominant sexual transmission. This becomes more important among the youths under study as they act as reservoirs for HIV. This study set out to determine the sexual behaviour of HIV-infected youths receiving ART care in Palapye District of Botswana, ascertain their attitudes towards safe sex and identify the factors that are associated with risky sexual behaviours.

### 4.1. Relationships and Disclosure

Little is known about the sexual behaviour of HIV-infected youths in Botswana. Like their HIV-negative counterparts, a significant proportion of HIV-infected youths desire to be or are already in relationships. Of all the respondents, 24% had ever had a boyfriend or girlfriend. This was, however, quite low compared with 41% which was obtained in a Ugandan study among HIV-infected youths of the same age group of between 15 and 19 years who were recruited through existing HIV/AIDS treatment, care and support centres [8,9,10]. More than half (64%) reported having ever had a boyfriend or girlfriend in a more recent study in central Uganda.

Among the respondents who had ever had boyfriends or girlfriends in this study, only 36 % had disclosed their HIV-positive status. These findings were comparable with previous studies which had HIV status disclosure rates ranging from 25% to 40% across different settings [22,35,36]. Disclosure has been shown to facilitate safe sex [37]. Qualitative findings suggest that disclosure of an HIV-positive status to partners made the youths vulnerable to abandonment or rejection and exposure or stigmatisation (7). This might explain the finding of consistently low disclosure rates, as reported in our study.

### 4.2. Sexual Preferences and Experiences

A significant proportion of HIV-infected youths are beginning to be involved in relationships and are exploring their sexuality by engaging in sexual intercourse. In terms of HIV status preference, this study revealed that whilst most of the youths (44%) also preferred an HIV-positive partner, a significant proportion (16%) preferred an HIV-negative partner. This figure is lower compared to the findings by Birungi et al. [8] where 37% of the respondents preferred HIV-negative partners. The majority of the participants preferred a partner who is also HIV-positive, a concept referred to as ‘serosorting’. Serosorting has been described by different scholars and even among HIV-infected youths and among men who have sex with men [38,39]. Although serosorting eliminates or makes the disclosure process easier for the youths, it addresses rejection and allows the youths to enjoy sexual relationships without worrying about infecting their partner with HIV, it is important to note that it might expose them to re-infection even with resistant HIV strains and other STIs.

Compared with similar cross-sectional studies among HIV-infected youths, the prevalence of sexual activity (having ever had sex) was quite low at 15.3%. Comparisons were difficult to make, especially in instances where research designs and approaches differed, notably the age groups that were included in the studies. In general, the prevalence of sexual activity increased with age with older youths being more likely to be sexually experienced compared with younger youths. Based on the literature, US and Ugandan studies revealed higher prevalence of sexual activity; 59% [14,36] and 45% [40]. However, a studies report [35,36] which acted as a prototype for Sub-Saharan Africa obtained a similarly low prevalence of sexual activity among HIV-positive youths of 14.9%. Differences with other African regions and continents can be explained by differences in cultural backgrounds and environmental differences since sexual behaviour is culture- and environment-specific.

### 4.3. Multiple Concurrent Partnerships

The finding that most of the ever-had sex youths (68%) had either no current sexual partner or only one sexual partner whilst 32% had multiple concurrent partnerships is quite comparable to the recent findings by Ankunda et al. [41], where 30% were found to be having multiple concurrent partnerships. Counter to this finding, some studies have reported even higher levels of multiple concurrent partnerships of up to 62% among HIV-positive youths [42].

### 4.4. Sexual Debut

The mean age of sexual initiation was 16.3 years and almost similar findings have been obtained by earlier researchers where HIV-positive youths on average initiated sexual intercourse at around ages 16.9 [36]. However, lower age at sexual initiation than what was reported for this study has also been reported elsewhere (14 years) among HIV-positive adolescents [38].

### 4.5. Unprotected Sexual Intercourse

Unprotected sexual intercourse was determined by the patterns of condom use among the sexually experienced youths. Findings from this study were no different from the findings of previous authors who reported consistently low levels of condom use among HIV-infected youths, with more than half (54–70%) of the sexually active youths reporting inconsistent condom use [22,36]. Although none of the sexually experienced youths reported that they never used condoms, almost three quarters (70%) reported inconsistent condom use, which is quite a disturbing finding considering the predominant sexual transmission of HIV. Although reasons or motivation for consistent condom use were not assessed in this study, some literature reported that prevention of pregnancy was the major reason for condom use whilst condom use to prevent HIV transmission and other STIs to a sexual partner and re-infection was low [43,44,45,46].

These high rates of unprotected sexual intercourse among HIV-infected adolescents might support the school of thought that as the HIV epidemic matures, HIV-positive youths feel that it is not their responsibility alone to protect their sexual partners from HIV infection but view it as a dual responsibility or rather the partner’s responsibility to protect themselves as well [38,43].

### 4.6. Attitudes towards Safe Sex

Studies assessing attitudes towards HIV/AIDS amongst youths generally show a good attitude towards HIV/AIDS [25,45,47]. The current study also revealed generally good attitudes towards safe sex. Another study which sought to describe knowledge, attitudes and practice on HIV/AIDS among university students in Ethiopia showed that those students who were knowledgeable and had favourable attitude had better preventive practices towards HIV/AIDS compared to students who were not knowledgeable and those who had unfavourable attitudes [36,48]. In this study, 46% of the youths would not disagree with having sex with a partner who was not prepared to use a condom. Many HIV youths believe that their partners have the responsibility of protecting themselves. If they refused to use the condom, the HIV-infected youths would continue with sexual intercourse even without disclosure. A study showed that this was likely to happen especially with a casual partner [27,28,29,30,36,43,49].

### 4.7. Factors Associated with Risky Sexual Behaviours

The selection of ever had sex as a risky sexual behaviour was based on the fact that abstinence, safer sex, delayed sexual initiation and improved condom use were some of the reported HIV/STI prevention interventions and peer norms had an effect on various factors [40,41].This, therefore, means that for youths within the age group under study (15–19 years), abstinence is a well-recognised safe sexual behaviour and, therefore, having ever had sex represents a risky sexual behaviour.

### 4.8. Alcohol, Smoking and Substance Use

There is consensus in the literature that alcohol and substance use are associated with risky sexual behaviours both among adults and youths [13]. Similar findings were reported in this study where both alcohol and substance use were significantly associated with risky sexual behaviour. Qualitative analyses described an increased desire to have sex and allowing an escape from responsibility as effects that were brought about by alcohol and drugs and contributed to risky sexual behaviours [4,5,6,43,48]. The results obtained suggest that there is a need to address alcohol and substance use among HIV-infected youths as a way of promoting safer sex practices.

### 4.9. Importance Attached to Religion

The literature suggests that a relationship exists between religion and sexual behaviour [50,51]. In a study conducted among university students, among those students who rated religion as less important, the probability of early sexual activity and having a high number of lifetime partners increased significantly. The same findings were obtained in the studies, where youths who did not consider religion to be important were almost six times more likely to report having ever had sex than youths who reported that religion was important to them.

### 4.10. Implication for Policy and Practice

Botswana Ministry of Health has developed and disseminated guidelines for the management of HIV-infected adolescents which attempt to offer a holistic approach and tailored service but is not being fully and uniformly applied. Most youths in this study had received information on sexual and reproductive health from schools. A significant proportion, however, preferred to receive such information from health care workers. The health care systems should, therefore, do more in terms of empowering these youths with information on sexual and reproductive health. This, however, requires that the healthcare workers and system be capacitated with the necessary systems and skills. Sensitisation and subsequent training of district focal persons on the guideline is recommended. Sex educators can be trained and given the task to educate youths both in schools and in healthcare settings about sexual behaviour. Teen clubs for HIV-positive youths should be revived and run by the districts and healthcare facilities with a consistent and dedicated budget. Boot camps can also be piloted. Youth friendly services and clinics should be offered in Palapye. These strategies should offer more secondary preventive messages to these youths using youth friendly services approaches. Interventions to empower these youths with skills to negotiate disclosure and consistent condom use should be strengthened using some of the above health care setting strategies. Youths who had ever discussed sex-related matters were likely to engage in safer sex than those that did not, hence parents, teachers and healthcare workers should be encouraged to discuss sexual and reproductive issues with these youths. Some youths in this study also desire to get sexual and reproductive health messages from the church. Churches through the district aids coordinating office should be sensitised and encouraged to teach HIV preventive methods for both HIV-positive and negative youths especially abstinence which fits in well within their context. More support is required to address alcohol and substance use among youths (HIV-positive youths included). As reservoirs of the HIV infection, any acts of unprotected sexual intercourse associated with alcohol use puts their sexual partners at risk. The above interventions should assist in building their capacity and confidence thereby empowering them to negotiate safer sex practices.

Schools Life Skills curriculum to include an equal content of secondary preventive strategies. It should also alert other youths, especially the HIV negative ones about the possibility of meeting an HIV-positive youth partner hence the need to invest in protecting themselves from HIV infection rather than giving the full responsibility to their partners. Condom promotion and distribution at healthcare facilities among HIV-positive youths should be promoted and monitored. Both male and female condoms should be promoted. Promotion of male circumcision would help reduce transmission where condoms were not used. Pre-exposure prophylaxis for stable discordant couples should be included in the HIV treatment policy/guidelines given the low condom use patterns observed in this study.

## 5. Limitations

This study should be interpreted considering its limitations. This study was a cross-sectional study therefore no causal inferences can be made from the associations that were found. Sexual behaviour described was based on self-reports by the respondents. The accuracy and quality of the knowledge was not assessed or measured. Therefore, the knowledge which the respondents claimed to have may not be the correct knowledge expected. Based on the sensitive nature of sexual behaviour and the research design which employed an interviewer administered questionnaire, the findings are subject to social desirability bias although several measures were put to limit this kind of bias. This study did not separate perinatally infected and behaviourally infected youths due to the anticipated complexity of the process due to inadequate records. Access to, and use of, patient records was not included in the methodology and when ethical clearance was sought for, hence recruitment into the study was not based on period enrolled in the ART program. However, most HIV-infected youths were, in fact, perinatally infected. The study was health care facility-based and was conducted in one district, therefore precludes generalisation to all youths in Botswana. In Botswana, the public ART programme is strictly for citizens, hence other youths, either local or foreign receiving ART from the private sector were excluded. Despite these limitations, the findings from this study contribute to the understanding of the sexual behaviour of HIV-infected youths and help address factors that were identified to be associated with risky sexual behaviour among this population group.

## 6. Conclusions

A significant proportion of HIV-infected youths in Palapye District are in relationships and are sexually active. Despite having generally good attitudes towards safe sex, preventive practices such as HIV status disclosure and condom use are poor. Among those youths that had ever had boyfriends or girlfriends, only approximately a third had disclosed their HIV status. The proportion of those that had ever had sex was comparable to figures that had been obtained regionally. Condom use was low. Alcohol and substance use, and attitude towards religion were associated with risky sexual behaviours. HIV remains a disease of public health interest and interventions both locally and at policy level are required to prevent onward transmission of HIV. HIV-infected youths should be empowered with skills to negotiate safe sex through different health promotion activities. There is therefore a need to invest in strategies that empower these HIV-positive youths to negotiate safe sex practice through disclosure and consistent condom use as a way of preventing transmission of HIV including super-infection and other STIs. This should result in improved sexual health among this population group.

## 7. Further Research

A study could further explain certain behaviours reported and explore secondary HIV prevention strategies. A review of literature on sexual behaviour of HIV-infected youths in Sub-Saharan Africa, especially in Botswana, reveals that this area is under-researched. There are gaps in the studies ascertaining attitudes of these youths towards safe sex. Further study in the field is recommended.

## Figures and Tables

**Table 1:** Socio-demographic characteristics of the participants.

Variable	Female	Male	Total
	n = 106 (56%)	n = 82 (44%)	n = 188 (100%)
Age distribution (n = 188)			
15	20 (18.9)	17 (20.7)	37 (19.7)
16	17 (16.0)	13 (15.9)	30 (16.0)
17	22 (20.7)	16 (19.5)	38 (20.2)
18	29 (27.3)	13 (15.9)	42 (22.3)
19	18 (17.0)	23 (28.1)	41 (21.8)
Educational Background (n = 188)			
Attended school	106 (100)	81 (98.8)	187 (99.5)
Never attended school	0 (0)	1 (1.2)	1 (0.5)
Highest level reached (n = 187)			
Primary	16 (15.1)	10 (12.2)	26 (13.8)
Secondary	88 (82.0)	67 (81.7)	155 (82.5)
Vocational	1 (0.9)	4 (4.9)	5 (2.7)
Certified	1 (0.9)	0 (00	1 (0.5)
Current schooling status (n = 188)			
In school	72 (67.9)	54 (65.9)	126 (67.0)
Out of school	34 (32.1)	28 (34.2)	62 (33.0)
Employment history (n = 62)			
Employed	7 (20.5)	7 (25.0)	14 (22.5)
Unemployed	27 (79.0)	21 (75.0)	48 (77.4)
Religion (n = 188)			
None	12 (11.3)	11 (13.4)	23 (12.2)
Christianity	91 (85.9)	69 (84.1)	160 (85.1)
Traditional	3 (2.8)	1 (1.2)	4 (2.1)
Moslem	0 (0.0)	1 (1.2)	1 (0.5)
Importance of religion (n = 188)			
Religion is important	84 (80.0)	63 (76.8)	147 (78.6)
Religion is not important	21 (20.0)	19 (23.2)	40 (21.4)
Caregiver status (n = 188)			
Father and mother	14 (13.2)	9 (11.0)	23 (12.2)
Father alone	4 (3.8)	6 (7.3)	10 (5.3)
Mother alone	40 (37.7)	32 (39.0)	72 (38.3)
Grandparents	26 (24.5)	14 (17.0)	40 (21.3)
Sibling	4 (3.8)	3 (3.7)	7 (3.7)
Other caregiver	17 (16.0)	14 (17.1)	31 (16.5)
Alone	1 (0.9)	4 (4.9)	5 (2.7)
Other habits (n = 188)			
Drinks alcohol	14 (13.3)	13 (15.9)	27 (14.4)
Smokes cigarettes	1 (0.9)	6 (7.3)	7 (3.7)
Substance use	0 (0.0)	4 (4.5)	4 (2.1)

**Table 2:** Description of basic reproductive health and social context of the participants.

Variable	Females	Males	Total
	n = 106	n = 86	n = 188
Who they talk to about important things (n = 188)			
Father	1 (0.9)	5 (6.1)	6 (3.2)
Mother	32 (30.2)	20 (24.4)	52 (27.7)
Grandparents	18 (17.0)	7 (8.5)	25 (13.3)
Sibling	8 (7.6)	7 (8.5)	15 (8.0)
Friends	16 (15.1)	13 (15.9)	29 (15.4)
Guidance teacher	9 (8.5)	1 (1.2)	10 (5.3)
Class teacher/other teachers	2 (1.9)	3 (3.7)	5 (2.7)
Keep things to self	7 (6.6)	16 (19.5)	23 (12.2)
Other relative	13 (12.3)	10 (12.2)	23 (12.2)
Ever discussed sex related matters (n = 188)			
Had discussed	71 (67.0)	43 (52.4)	114 (60.6)
Never discussed	35 (33.0)	39 (47.6)	74 (39.4)
How easy it was to discuss sex related matters (n = 115)			
Easy	26 (32.5)	12 (27.3)	38 (30.7)
Average	30 (37.5)	13 (29.6)	43 (34.7)
Difficult	24 (30.0)	19 (43.2)	43 (34.7)
Reproductive health knowledge (n = 118)			
Yes	90 (84.9)	73 (89.0)	163 (86.7)
No	16 (15.1)	9 (11.0)	25 (13.3)
Important Source of information			
Mother	10 (9.4)	7 (8.5)	17 (9.0)
Father	0 (0.0)	7 (8.5)	7 (3.7)
Sibling	1 (0.9)	3 (3.7)	4 (2.1)
Friends	4 (3.8)	2 (2.4)	6 (3.1)
Healthcare workers	19 (17.9)	7 (8.5)	26 (13.8)
Teachers	37 (34.9)	24 (29.3)	61 (32.5)
Books/magazines	6 (5.7)	6 (7.3)	12 (6.4)
Media (TV, radio, movies)	11 (10.4)	12 (14.6)	23 (12.2)
Other relatives	1 (0.9)	0 (0.0)	1 (0.5)
Church	6 (5.7)	4 (4.9)	10 (5.3)
Internet	4 (3.8)	6 (7.3)	10 (5.3)
Preferred source of information			
Mother	11 (10.4)	10 (12.2)	21 (11.2)
Father	1 (0.9)	3 (3.7)	4 (2.1)
Sibling	3 (2.8)	0 (0.0)	3 (1.6)
Friends	4 (3.8)	2 (2.4)	6 (3.2)
Healthcare workers	38 (35.9)	26 (31.7)	64 (34.0)
Teachers	14 (13.2)	10 (12.2)	24 (12.8)
Books/magazines	9 (8.5)	10 (12.2)	19 (10.1)
Media (TV, radio, movies)	17 (16.0)	16 (19.5)	33 (17.6)
Other relatives	2 (1.9)	0 (0.0)	2 (1.1)
Church	7 (6.6)	5 (8.5)	12 (6.4)
Preferred partner HIV status			
HIV-negative	17 (16.2)	14 (17.1)	31 (16.6)
HIV-positive	53 (50.5)	30 (36.6)	83 (44.4)
No preference, any status	35 (33.3)	38 (46.3)	73 (39.0)

**Table 3:** Self-reported sexual behaviour among the HIV-infected youths by gender.

Variable	Females	Males	Total
	n = 106	n = 82	n = 188
Ever had a boy/girlfriend (n = 188)			
Yes	27 (25.5)	18 (22.0)	45 (23.9)
No	79 (74.5)	64 (78.050	143 (76.1)
Current boyfriend (n = 188)			
Yes	20 (18.9)	13 (15.9)	33 (17.6)
No	86 (81.1)	69 (84.2)	155 (82.5)
HIV status disclosure to boy/girlfriend (n = 45)			
Yes	10 (38.5)	5 (26.3)	15 (33.3)
No	16 (61.5)	14 (73.7)	30 (66.7)
Ever had sex (n = 188)			
Yes	10 (16.0)	12 (14.6)	29 (15.4)
No	89 (84.0)	70 (85.4)	159 (84.6)
Age at first sex (n = 29)			
≤16 years	6 (35.3)	4 (33.3)	10 (34.5)
>16 years	11 (64.7)	8 (66.7)	19 (65.5)
First sex planned (n = 29)			
Yes	5 (29.4)	3 (25.0)	8 (27.6)
No	12 (70.6)	9 (75.0)	21 (72.4)
Condom use at first sex (n = 29)			
No	11 (64.7)	8 (66.7)	19 (65.5)
Yes	6 (35.3)	4 (33.3)	10 (34.5)
Use of other contraception at first sex (n = 29)			
Yes	3 (17.7)	1 (8.3)	4 (13.8)
No	14 (82.4)	11 (91.7)	25 (86.2)
Had sex during the past 3 months (n = 29)			
Yes	9 (52.9)	7 (58.3)	16 (55.2)
No	8 (47.1)	5 (41.7)	13 (44.8)
Condom use at last sex (n = 29)			
Yes	9 (52.9)	3 (25.0)	12 (41.4)
No	8 (47.1)	9 (75.0)	17 (58.6)
Under alcohol influence at last sex (n = 28)			
Yes	8 (47.1)	8 (66.7)	16 (55.2)
No	9 (53.0)	4 (33.3)	13 (44.8)
Number of sexual partners (n = 28)			
0	3 (18.8)	1 (8.3)	4 (14.3)
1	8 (50.0)	7 (58.3)	15 (53.6)
2	4 (25.0)	4 (33.3)	8 (28.6)
4	1 (6.3)	0 (0.0)	1 (3.6)
Condom use practice (n = 29)			
I always use condoms	6 (35.3)	3 (25.0)	9 (31.0)
I sometimes use condoms	11 (64.7)	9 (75.0)	20 (69.0)
Pregnancy or having impregnated a girl (n = 29)			
Yes	7 (41.2)	2 (16.7)	9 (31.0)
No	10 (58.8)	10 (83.3)	20 (69.0)

**Table 4:** Distribution of respondents by reported attitude towards safe sex.

No.	Attitude Indicator	Scale			
		Agree	Disagree	Not Sure	Total
1 [25]	It is alright for unmarried youths to be in relationships	130 (69.2)	43 (22.9)	15 (8.0)	188 (100)
2 [26]	I believe that even HIV-positive unmarried youths can be in relationships	142 (75.5)	30 (16.0)	16 (8.5)	188 (100)
3 [27]	I believe it is not alright for youths of the opposite sex to hug, touch and kiss	62 (33.0)	105 (55.9)	21 (11.2)	188 (100)
4 [28]	I believe there is nothing wrong with unmarried youths having sexual intercourse as long as they love each other	99 (52.7)	68 (36.2)	21 (11.2)	188 (100)
5 [29]	I would refuse to have sex with someone who is not prepared to use a condom	78 (41.5)	87 (46.3)	23 (12.2)	188 (100)
6 [30]	One night stands are ok	21 (11.2)	146 (77.7)	21 (11.2)	188 (100)
7 [27]	It is embarrassing for someone like me to buy or obtain condoms	60 (31.9)	104 (55.3)	24 (12.8)	188 (100)
8 [31]	If a girl suggested using condoms to her partner, it means she doesn’t trust him	38 (20.2)	121 (64.4)	29 (15.4)	188 (100)
9 [32]	Condoms can protect my sexual partner from getting HIV	136 (72.3)	38 (20.2)	14 (7.5)	188 (100)
10 [33]	I would prioritise protecting my sexual partner from being infected with HIV	157 (83.5)	19 (10.1)	12 (6.4)	188 (100)
11 [34]	I would prioritise protecting myself from being infected with a new HIV virus or other sexually transmitted infections.	171 (91.0)	11 (5.9)	6 (3.2)	188 (100)

**Table 5:** Comparison of the proportion of selected socio-demographic characteristics, reproductive health and social context and sexual behaviour of HIV-infected youths.

Socio-Demographic Characteristics and Reproductive Health and Social Context Variables		Responses			*p*-Value
	Ever Had Sex				
		No	Yes	Total	
Age	≤17 years	96	8	104	
	>17 years	63	21	84	0.001
Current school status	In school	42	18	60	
	Out of school	117	11	128	0.000
Importance attached to religion	Important	139	8	147	
	Not important	20	20	40	0.000
Alcohol	No	150	10	160	
	Yes	8	19	27	0.000
Smoking	No	158	23	181	
	Yes	1	6	7	0.000
Substance use	No	158	26	184	
	Yes	1	3	4	0.001
Sexual reproductive knowledge	No	13	12	25	
	Yes	146	17	163	0.000
	Condom use practice				
		Always	Sometimes		
Importance attached to religion	Important	5	3	8	
	Not important	3	17	20	0.012
Alcohol	No	7	3	10	
	Yes	2	17	19	0.001
Ever discussed sex related matters	No	0	14	14	
	Yes	9	6	15	0.000
Sexual reproductive knowledge	No	0	12	12	
	Yes	9	8	17	0.002
	HIV status disclosure				
		Yes	No		
Ever discussed sex related matters	No	16	3	19	
	Yes	14	12	26	0.033
Sexual reproductive knowledge	No	12	1	13	
	Yes	18	14	32	0.020

**Table 6:** Logistic regression for having ever had sex.

Socio-Demographic Characteristics and Reproductive Health and Social Context Variables		*p* Value	OR	CI
Age	≤17 years >17 years	0.623	1.4	0.35–5.78
Current school status	In school Out of school	0.996	1.0	0.25–4.01
Importance attached to religion	Important Not important	0.005	5.9	1.72–20.27
Alcohol	No Yes	0.000	12.5	3.06–51.37
Smoking	No Yes	0.846	1.30	0.09–18.86
Substance use	No Yes	0.390	5.77	0.11–313.76

OR—Odds Ratio, CI—95% Confidence Interval.

## Data Availability

Data can be made available on reasonable request to the authors.
